# Factors influencing adherence to anti-retroviral therapy in amazonian indigenous people living with HIV/AIDS

**DOI:** 10.1186/s12889-023-15362-y

**Published:** 2023-03-15

**Authors:** Félix Valenzuela-Oré, Yolanda Angulo-Bazán, Lucy D. Lazóriga-Sandoval, Norma L. Cruz-Vilcarromero, Cecilia R. Cubas-Sagardia

**Affiliations:** 1 Centro Nacional de Salud Intercultural, Instituto Nacional de Salud Lima, Perú; 2 Dirección Regional de Salud Amazonas, Chachapoyas, Perú

**Keywords:** Adherence, HIV-AIDS, Indigenous populations

## Abstract

**Background:**

Indigenous communities in Peru has been historically affected by high mortality rates attributable to HIV-AIDS infection, associated with a low access to health services, and socio-cultural barriers. In this context, the study aimed to describe factors associated with antiretroviral treatment adherence in people from Awajun and Wampis indigenous communities, living with HIV-AIDS in a Peruvian Amazonian region.

**Methods:**

A cross-sectional study was completed with a consecutive sample of people from indigenous communities (Awajun or Wampis) living with HIV, who were receiving antiretroviral treatment for at least the last three months. Participants were recruited between October 1 and December 30, 2021, from four districts of Bagua and Condorcanqui provinces in the Amazonian region. An ad-hoc questionnaire was used to collect information about demographic, economic, and socio-cultural factors and access to health services. The Simplified Medication Adherence Questionnaire (SMAQ) was used to evaluate adherence to antiretroviral therapy. Multivariate logistic regression analysis with backward stepwise was performed to explore factors that might influence adherence.

**Results:**

Of the 208 participants, 28.8% reported complete adherence to antiretroviral treatment. The multivariate logistic regression showed that occupation (aPR: 1.86; 95%CI 1.15–3.02), economic income (aPR: 0.64; 95%CI 0.41–0.99), and adverse reactions to antiretroviral therapy (aPR: 0.36; 95%CI 0.18–0.70) were related to complete adherence to medication.

**Conclusion:**

Only a third of participants reported complete adherence to antiretroviral therapy. Factors associated with adherence to antiretroviral medication were related to socioeconomic conditions and adverse reactions to the therapeutic scheme. Interventions to improve adherence in indigenous people living with HIV should consider these factors in order to develop effective implementation strategies.

**Supplementary Information:**

The online version contains supplementary material available at 10.1186/s12889-023-15362-y.

## Background

The introduction of highly active antiretroviral therapy (ART) has converted HIV infection from a terminal disease to a treatable chronic disease. However, even though ART improves the clinical, immunological, and viral response, it requires maintaining a high level of adherence to the prescribed therapy due to the danger of an increase in viral activity and the development of resistance to these drugs [1]. In recent years, non-adherence to ART has become a complex public health problem with many associated factors that are related to the social, economic, and cultural context of people living with HIV. The World Health Organization (WHO) suggests that these factors can be classified into four dimensions: characteristics of the therapeutic scheme, patients’ characteristics, the relationship between healthcare providers and patients, and the proper characteristics of health systems. In the patient’s characteristics, one of the most important factors are the belief systems about health and disease that people should have due to their sociocultural context [2].

Indigenous populations are one of the groups most affected by the consequences of HIV infection and limited access to healthcare. Previous research has found that indigenous populations have higher rates of incomplete adherence or complete discontinuation of ART compared to the general population, possibly due to factors related to their cultural uniqueness and their views or interpretations about disease and conventional treatments, as well as social aspects such as geographical barriers, access and availability of monitoring, etc [3–5]. Thus, this situation suggests that public health strategies to control the transmission of HIV in indigenous populations should be developed using a multilevel approach based on human rights and integrating traditional practices that can help reduce vulnerability to transmission [6].

Peru is one of the Latin American countries with the largest number of indigenous populations in the region. According to official records, in 2017, there were 44 indigenous populations in 2703 communities at the national level [7]. On the other hand, the epidemiology of the HIV/AIDS infection in Peru still has characteristics of a concentrated epidemic, with an estimated prevalence of 0.3% in adults older than 15 years, an incidence rate of 0.20 per 1000 inhabitants, with approximately 1000 deaths per year [8, 9]. Awajun and Wampis are Amazonian indigenous populations, who represent 35.8% of the total indigenous population affected by sexually transmitted diseases, including HIV infection [7].

The Amazonas Region concentrates 13.4% of the Peruvian indigenous communities, with an important presence of Awajun and Wampis populations. According to statistics from the Regional Health Direction, the districts with the most presence of Awajun and Wampis populations report more than 70% of HIV infection cases from the entire region [10]. There is some preliminary evidence which suggests that the conception and social representations of health and disease, especially in the case of HIV infection in people belonging to these indigenous populations, strongly influence the behaviors that they finally present before the health interventions that the conventional health system tries to impose [11–13]. However, there has been no evidence found related to factors influencing ART adherence in these populations. Therefore, this study aimed to describe factors associated with antiretroviral treatment adherence in people from Awajun and Wampis indigenous communities living with HIV-AIDS in four districts from a Peruvian Amazonian region.

## Methods

### Study design and participants

This was a cross-sectional study that included people living with HIV, who were over 18 years old, from Awajun or Wampis communities, who were living in the districts of Santa María de Nieva, El Cenepa, Río Santiago, and Imaza (Amazonas Region-Condorcanqui and Bagua provinces), and who had an ART prescription for at least three months before the survey application. People who had not received ART in the last three months because of supply or logistical problems, previous failure of ART or ART resistance, or who could not express themselves or did not sign the informed consent form were excluded from the study. Participants were included using a non-probabilistic sampling by convenience.

Participants were recruited between October 1 and December 30, 2021. In this context, according to medical records reviewed before the survey application, there were 292 people living with HIV in the four districts that would be evaluated in the study. The cases were distributed as follows: 82 in Santa Maria de Nieva district, 104 in Cenepa district, 42 in Rio Santiago district, and 64 in Imaza district.

The study was approved by the Ethical Review Board from the Instituto Nacional de Salud-Peru (RD N° 065-2020-DG-OGITT-OPE/INS). An informed consent process was applied in the local language, explaining the study’s aims and participation implications to the participants. Only participants who signed the informed consent form were included in the study.

### Assessment of ART adherence

ART adherence in last three months were evaluated with the Spanish adapted version of Simplified Medication Adherence Questionnaire (SMAQ) [14]. The SMAQ has been used in previous research [15] and as a monitoring instrument in health public services in Peru [16]. The questionnaire has six items with dichotomous answers (Yes/No):

1. Do you ever forget to take your medication?
2. Did you always take your medications at the right time?
3. Do you ever stop taking your medicine if you feel sick?
4. Did you forget to take your medication over the weekend?
5. I have not taken more than two doses in the last week.
6. Did you forget to take the medicine more than two days, in the last three months?

It was considered as a “non-adherent” anyone who had a positive answer to any of the six questions. For the last two questions, a positive answer was considered when the participant indicated forgetting to take medication for two or more doses in the last week, or for more than two days in the last three months.

### Assessment of possible associated factors

Data related to personal characteristics (age, gender, civil status, educational level, occupation, religion, monthly income, alcohol, tobacco or drug consumption, family members and migration), clinical features (disease time, risk population, opportunistic infections), factors related to ART (therapeutic scheme, adverse events), sociocultural factors (disease conceptualization, medicinal plant use, attention by traditional medicine agent, familiar support, social support, stigmatization, and discrimination) and healthcare access (healthcare insurance, time to the nearest health centers, transport expenses to health centers, expenses in ART medications) were evaluated by an ad-hoc format developed by the research team based on a previous literature review (view Supplementary Material) [13]. That instrument was previously validated by experts.

Data about social support and familiar support were collected with a Likert scale with five categories (very well, well, regular, bad, very bad), according to the concept proposed by the national framework of integral attention of people living with HIV, developed by the Ministry of Health (Peru) [17]. In the same way, data about clinical features and ART schemes were collected according to the recommendations of the same document (view Supplementary Material). Additionally, information about ART features was corroborated with a review of medical records in local primary care centers.

### Data collection procedures

Previously, before conducting surveys, the researchers collaborated with the regional health direction, health networks, and primary care centers. They were informed about the study and were asked for their cooperation in providing information about the medical records and locations of people living with HIV from Awajun and Wampis communities.

On the other hand, the researchers made official communications with the provincial municipality and communal authorities (“apus”), who signed the collective informed consent form. At this point, eight local health professionals with experience in healthcare for these indigenous communities were identified and trained to collect data using validated instruments.

The participants were recruited by appointments at the nearest primary health care center, where trained professionals identified potential participants who met the selection criteria. This process was conducted under the supervision of the representative of the regional health direction and the research team.

Each primary health care center was equipped with an appropriate space to ensure privacy and build confidence with potential participants. If any participant wanted to be surveyed in their home, a visit was scheduled, ensuring privacy and confidentiality. The local language was used during data collection to ensure the quality of the information.

### Statistical analysis

A codified database was created in Microsoft Excel 2016 by two independent typists who were not part of the research team. Registries with inconsistent or missing data were excluded from statistical analysis. Descriptive measures (frequencies, percentages, means or medians, and standard deviation/interquartile range) were calculated according to the nature of variables (qualitative or quantitative).

After that, a bivariate analysis was performed to evaluate the association between possible factors (personal characteristics, clinical features, factors related to ART, sociocultural factors, and healthcare access) and adherence to ART. Fisher’s Exact Test or Chi Square Test were used for qualitative variables; and Student’s t-test for independent data or Mann-Whitney U for quantitative variables.

A multivariate analysis was developed through multiple logistic regression controlling for age, gender, and clinical stage and considering ART adherence as a dichotomous outcome (Complete/Incomplete). Crude and adjusted prevalence ratios (PR) with their corresponding 95% confidence intervals (CI 95%) were calculated to evaluate the magnitude of found associations. A backward stepwise selection was performed, considering a significant level of 0.2 for inclusion in the multivariate model.

Statistical analyses were performed using R 4.0.5, R Foundation for Statistical Computing, Vienna, Austria. A *p*-value less than 0.05 was considered statistically significant.

## Results

### General characteristics

The study collected information from 208 participants from 55 indigenous Awajun or Wampis communities and 31 primary health care centers. The median age was 25 (22–32) years old, with 57.2% males and 88.9% from Awajun population. Most of them were single (43.3%) or reported living with a partner without being married (45.2%), with a median income of US$ 73.7 (24.6-122.8), although 47.6% reported not having any income. 174 participants were living in a family (83.7%), although 21 participants reported living alone and far away from family, and 13 (6.2%) alone but close to family. 52.4% mentioned that they did not usually travel to cities.

Most of the participants mentioned that they were not included in a risk population (80.3%), but 16.8% reported being included in the group of “men who have sex with men” (MSM). There was an approximate median disease time of 3.6 years, with 24% of participants who reported having at least one opportunistic condition at some point. Four cases of tuberculosis-HIV co-infection were found.

73.1% of participants mentioned that they were using only ART medications, but 18.8% reported using ART concomitant with medicinal plants. The first-line therapeutic scheme was the most used (39.9%) with a median treatment time of 2.8 years. 33.2% of respondents reported having adverse effects during the ART.

When they were asked about their conceptualization of the disease, the majority (82.7%) referred to a cause consistent with current knowledge about this disease (sexual transmission or blood transfusion); however, 13% of participants referred to concepts of the disease related to magical-religious origins. Most of the participants (51.0%) reported receiving poor family support, and 26.9% perceived very poor family support. Additionally, 84.1% reported perceiving stigmatization in their health facility due to their condition and 86.1% mentioned having been discriminated against by being denied care in health services due to their illness.

Most of the participants reported having state insurance provided by the Ministry of Health - Seguro Integral de Salud (90.9%), but 4.8% mentioned not having any health insurance. Additionally, the median time to travel to the primary care center was 10 (5–30) minutes with a maximum of five days approximately. 41.3% of participants reported any type of expense for traveling to the health center, and 30.8% mentioned making any expense to buy HIV treatment. Additional information about the characteristics of participants is explained in Table 1.

**Table 1:** General characteristics of indigenous people living with HIV from Condorcanqui and Bagua provinces - Amazonas Region

Characteristics	n (%)
**Age (years-old)** ^a^	25 (22–32)
**Gender**	
Female	89 (42.8)
Male	119 (57.2)
**District** ^c^	
1	76 (36.5)
2	74 (35.6)
3	38 (18.3)
4	20 (9.6)
**Ethnic group**	
Awajun	185 (88.9)
Wampis	20 (9.6)
Others	3 (1.4)
**Marital status**	
Married	10 (4.8)
Single	90 (43.3)
With partner (non-married)	94 (45.2)
Divorced	6 (2.9)
Widower	8 (3.4)
**Educational level**	
Without studies	10 (4.8)
Primary level	44 (21.2)
Secondary level	130 (62.5)
Superior studies	24 (11.5)
**Occupational status**	
Other jobs	91 (43.8)
Agriculture	60 (28.8)
Employed	26 (12.5)
Student	20 (9.6)
Laborer	10 (4.8)
Hunting or fishing	1 (0.5)
**Religion**	
Evangelic	86 (41.3)
Non-religious	38 (18.3)
Christian	34 (16.3)
Catholic	22 (10.6)
Israelite religión	6 (2.9)
Other	22 (10.6)
**Income (S/.)** ^a^	300 (100–500)
Yes	109 (52.4)
Not	99 (47.6)
**Alcohol use** ^b^	
Yes	47 (22.6)
Not	161 (77.4)
**Tobacco use** ^b^	
Yes	16 (7.7)
Not	192 (92.3)
**Drug or illicit substance use** ^b^	
Yes	3 (1.4)
Not	205 (98.6)
**People who are living with**	
With family	174 (83.7)
Alone and far to the family	21 (10.1)
Alone but close to the family	13 (6.2)
**Travelling frequently to the cities?**	
Yes	99 (47.6)
Not	109 (52.4)
**Risk group**	
MSM	35 (16.8)
TG	4 (1.9)
TS	2 (1.0)
PG	167 (80.3)
**Disease time (days)**	1324.5 (417.8–1890.0)
**Opportunistic diseases**	
Yes	50 (24.0)
Not	158 (76.0)
**Clinical stage (CS)**	
CS1	196 (94.2)
CS2	9 (4.3)
CS3	3 (1.4)
CS4	0 (0.0)
**TB-HIV coinfection**	
Yes	4 (1.9)
Not	204 (98.1)
**Type of treatment**	
Only ART	152 (73.1)
Only with medicinal plants	9 (4.3)
ART and medicinal plants	39 (18.8)
Self-medication	8 (3.8)
**Therapeutic scheme**	
First line	83 (39.9)
Tenofovir based	78 (37.5)
Abacavir based	0 (0.0)
Zidovudine based	3 (1.4)
Does not know/ Not answer	44 (21.2)
**Therapeutic time (days)**	1028 (361–1687)
**Adverse events to ART**	
Yes	69 (33.2)
Not	139 (66.8)
**Health insurance**	
Not have	10 (4.8)
SIS	189 (90.9)
EsSalud	9 (4.3)
**Transportation expenditures** ^a^	
Not	122 (58.7)
Yes	86 (41.3)
**Treatment expenditures**	
Not	144 (69.2)
Yes	64 (30.8)

*MSM *Men who have sex with men, *TG *Transgender people, *SW *Sexual workers, *GP *General population, *TB *Tuberculosis, *HvB *Hepatitis B virus

^a^Results in median (interquartile range). ^b^Use once per week at least. ^c^The name of districts has not to be shown in order to keep confidentiality of participants

A complete ART adherence was evidenced in 28.8% of participants (42.3% in Awajun people and 25% in Wampis). The dimensions with better accomplishment were taking medication at the indicated hour (76.0%) and not stopping to take medication if they felt sick (74.5%). In contrast, the dimension with the least accomplishment was forgetting to take medication (53.8%). The results by SMAQ dimensions are expanded in Table 2.

**Table 2:** Adherence to ART in indigenous people from Condorcanqui and Bagua-Amazonas

Items	N	%
**Do you ever forget to take your medication?**		
Yes	112	53.8
Not	96	46.2
**Did you always take your medications at the right time?**		
Yes	158	76.0
Not	50	24.0
**Do you ever stop taking your medicine if you feel sick?**		
Yes	53	25.5
Not	155	74.5
**Did you forget to take your medication over the weekend?**		
Yes	69	33.2
Not	139	66.8
**I have not taken more than two doses in the last week**		
None	118	56.7
1–2	46	22.1
3–5	9	4.3
6–10	2	1.0
More than 10	33	15.9
**Did you forget to take the medicine more than two days, in the last three months?**		
None	132	63.5
Less than two days	18	8.7
Two or more days	44	21.2
Not known /not answered	14	6.7

### Bivariate analysis

A significant statistical association was found between ART adherence and marital status (*p* = 0.011), alcoholism (*p* < 0.001), adverse reactions to ART (*p* < 0.001), and reporting stigma (*p* = 0.021) or discrimination (*p* = 0.003). The complete results of the bivariate analysis are shown in Table 3.

**Table 3:** Bivariate analysis of associated factors to ART adherence in indigenous population from Condorcanqui and Bagua provinces-Amazonas

Characteristics	Adherence		*p*-value^b^
	Complete	Incomplete	
	(*n* = 60)	(*n* = 148)	
**Age (years old)**	26 (22–32)	25 (22–32)	0.659^a^
**Gender**			
Male	88 (73.9)	31 (26.1)	0.354
Female	60 (67.4)	29 (32.6)	
**Marital status**			
Married	3 (30.0)	7 (70.0)	
Single	20 (22.2)	70 (77.8)	
With partner (non married)	37 (39.4)	57 (60.6)	0.011
Divorced	0 (0.0)	6 (100.0)	
Widower	0 (0.0)	8 (100.0)	
**With occupation**			
Not	22 (24.2)	69 (75.8)	0.218
Yes	38 (32.5)	79 (67.5)	
**With any regular income**			
Not	35 (35.4)	64 (64.6)	0.065
Yes	25 (22.9)	84 (77.1)	
**Alcohol use**			
Not	56 (34.8)	105 (65.2)	< 0.001
Yes	4 (8.5)	43 (91.5)	
**Tobacco use**			
Not	58 (30.2)	134 (69.8)	0.161
Yes	2 (12.5)	14 (87.5)	
**Drug or illicit substance use**			
Not	60 (29.3)	145 (70.7)	0.558
Yes	0 (0.0)	3 (100.0)	
**Disease time (days)**	1389.5 (383.8- 1883.0)	1297.0 (443.2- 1929.8)	0.953^a^
**Opportunistic diseases**			
Not	51 (32,3)	107 (67,7)	0,072
Yes	9 (18,0)	41 (82,0)	
**Clinical stage**			
CS1	57 (29,1)	139 (70,9)	1,000
CS2	2 (22,2)	7 (77,8)	
CS3	1 (33,3)	2 (66,7)	
**Adverse events related to ART**			
Not	53 (38,1)	86 (61,9)	< 0,001
Yes	7 (10,1)	62 (89,9)	
**Disease conceptualization**			
Conventional	50 (29.1)	122 (70.9)	1.000
Magical or religious	10 (27.8)	26 (72.2)	
**Stigma**			
Not	4 (12.1)	29 (87.9)	0.021
Yes	56 (32.0)	119 (68.0)	
**Discrimination**			
Not	2 (6.9)	27 (93.1)	0.003
Yes	58 (32.4)	121 (67.6)	

^a^U Mann-Whitney Test

^b^Exact Fisher Test

### Multivariate analysis

When the multivariable analysis was performed, it was observed that the interactions of age with the possible associated factors did not have statistically significant results, so it was decided to remove this variable as an adjustment variable, however, age was retained in the multivariate model.

Finally, it was observed that having an occupation was associated with a greater probability of having complete adherence (aPR 1.86; CI 95% 1.15–3.02), while having any monthly income (aPR 0.64; CI 95% 0.41–0.99) and reporting adverse effects (aPR 0.36; CI 95% 0.18–0.70) were related to a lower probability of having complete adherence. Although a significant effect of clinical stage was evidenced, it was quite small and with wide confidence intervals due to the small sample size in the study categories. Complete results of crude and adjusted models are shown in Table 4.

**Table 4:** Multivariate model of associated factors to ART adherence in indigenous population from Condorcanqui and Bagua provinces-Amazonas

Characteristics	Univariate		Multivariate^a^	
	PR (CI 95%)	*p*-value	PR (CI 95%)	*p*-value
**Age (years-old)**	1,01 (0,98 − 1,04)	0,564	1,00 (0,98 − 1,02)	0,981
**Gender**				
Male	Ref		Ref	
Female	1,25 (0,82 − 1,92)	0,304	1,36 (0,84 − 2,18)	0,207
**Marital status**				
Without partner	Ref		Non included	
With partner	2,00 (1,26 − 3,18)	0,003		
**With occupation**				
Not	Ref		Ref	
Yes	1,34 (0,86 − 2,10)	0,198	1,86 (1,15 − 3,02)	0,012
**Having any income (S/.)**				
Not	Ref		Ref	
Yes	0,65 (0,42 − 1,00)	0,052	0,64 (0,41 − 0,99)	0,045
**Alcohol use**				
Not	Ref		Ref	
Yes	0,24 (0,09 − 0,64)	0,004	0,46 (0,18 − 1,13)	0,091
**Tobacco use**				
Not	Ref		Non included	
Yes	0,41 (0,11 − 1,54)	0,189		
**Drug or illicit substance use**				
Not	Ref		Non included	
Yes	0,00 (0,00–0,00)	< 0,001		
**Disease time (days)**	1,00 (1,00–1,00)	0,790	Non included	
**Opportunistic diseases**				
Not	Ref		Non included	
Yes	0,56 (0,30 − 1,05)	0,071		
**Adverse events to TAR**				
Not	Ref		Ref	
Yes	0,27 (0,13 − 0,55)	< 0,001	0,36 (0,18 − 0,70)	0,003
**Disease conceptualization**				
Conventional	Ref		Non included	
Magical or religious	0,96 (0,54 − 1,70)	0,877		
**Use of medicinal plants**				
Not	Ref		Ref	
Yes	0,44 (0,21 − 0,91)	0,026	0,53 (0,27 − 1,01)	0,054
**Stigma**				
Not	Ref		Non included	
Yes	2,64 (1,03–6,80)	0,044		
**Discrimination**				
Not	Ref		Ref	
Yes	4,70 (1,21 − 18,25)	0,025	3,52 (0,92 − 13,41)	0,065

^a^Controlled by gender and clinical stage with a backward stepwise selection, considering a significance level of 0.2 to select variables

## Discussion

This research found that 28.8% of surveyed people had complete adherence to ART. In urban contexts in Peru, previous research has found ART adherence levels of 65.1%, which is greater than the values found in this study [16]. Similarly, Omosanya et al., (2013) found an ART adherence of 88% in people living with HIV from a rural tertiary medical center in Nigeria, which suggests that area of residency is not necessarily a influencing factor on increasing adherence [18]. In contrast, Chavarry-Ysla et al., (2020) found an ART adherence of 33% in people from the Peruvian ethnic group Kusu-Pagata, which is similar to this study [19]. This is consistent with other studies in Latin American indigenous communities with adults living with HIV, which report adherence levels between 20–50%. [20].

No association was found between ART adherence and personal characteristics. However, the profile is consistent with previous research in indigenous people living with HIV, with the exception of the gender distribution. A previous publication about indigenous people from Bolivia living with HIV reports a 5:3 ratio between males and women [21], but findings from another publication that reports the situation in eight Amazonian ethnic groups in Brazil shows a distribution of 60% of women affected by HIV infection [22].

An association between having an occupation and the probability of having complete ART adherence was found. More than 80% of surveyed people reported living with their families and approximately 40% reported having occasional jobs. These social factors are closely related to poverty conditions in the Condorcanqui province, which is considered among the top 10 poorest provinces in the country according to the INEI’s 2018 Monetary Poverty Map [23]. The occupational status has been proved to be a crucial element in improving ART adherence in people, regardless of the income classification of the countries they are from [24, 25]. Previous research has hypothesized that the occupational status could be related to more careful behaviors related to improving self-care, in order to continue with productive activities that contribute to maintaining life quality and health status [25, 26].

In contrast, factors such as having any monthly income or reporting adverse effects were related to not having complete adherence. This study found that, in people who receive a salary, the median income was lower than the basic salary in Peru. Although previous research has found some differences when using indicators such as the Gini coefficient to measure variables like “income inequality” [27], or when individual income is measured through ordinal scales with more than two categories [28]. It should be noted that the result is not a robust measure and the confidence intervals of prevalence ratios were close to the unit. Therefore, an extremely careful interpretation is highly suggested and the possibility of a non-measured confounding effect as a cause of the results should not be dismissed.

On the other hand, previous evidence has suggested that the presence of any adverse effect related to ART is strongly related to a decrease in adherence. For example, a systematic review developed by Al-Dakkak et al., (2013) explains that specific adverse events such as fatigue, taste disturbance, or nausea were significantly related to having lesser adherence, in comparison to people who do not have other types of adverse events [29]. In this research, data about adverse events were collected in a general way, without specifications, so it is suggested that future research develops more detailed instruments in order to improve the quality of data related to this variable.

Additionally, it is important to consider other factors that influence the relationship between adverse effects and adherence, such as the response to access to health services when these effects occur. Local qualitative reports suggest that indigenous people who had adverse effects had a higher probability of abandoning the treatment, due to lack of transportation to reach health services, lack of medicines and the consequent out-of-pocket expense to assume treatment of adverse effects, and lack of health professionals in primary level establishments. This, added to personal factors such as the nutritional status of patients and cultural factors such as the belief that medicines “cause harm,“ generate mistrust towards health services in these populations [30].

Is important to be highlight that approximately 13% of participants referred to a disease conceptualization related to magical or religious origins. Previous qualitative research has found some references, particularly in awajun groups, about the “disease” (HIV) as a consequence of sorcery (“*brujeria*”) on some members of the community [31]. In this context, it is important to clarify that this sorcery or harm (translated in Spanish as “daño”) is only one of the numerous terms related to the complex universe of affection relationships in the awajun cosmovision of disease [32]. For example, previous research referred to local expressions among Awajun people as “an unprecedented proliferation of *tunchis*” (the first mythological shaman to live at the bottom of the waters), which makes the evils or harm become something similar to the symptoms of HIV-AIDS, as a result, they get sick and are never saved [32]. This could explain some previous findings related to not being afraid of being infected with HIV by sexual transmission, favoring its spread and possibly limiting adherence to treatmentf [19].

Likewise, people reported that 18.8% combine ART with medicinal plants, and 4.4% take only medicinal plants as a treatment for their disease. Although the consumption of medicinal plants is a common behavior in chronic patients, especially in people living with HIV [11, 19], among indigenous people, this could be considered as a ritual intervention with the use of specific types of plants (“*plantas aliadas*”) and mobilization of cosmological ambits, which permit the restoration of physical folds and soul (“*dietar*”) [32].

No association between the other sociocultural factors and ART adherence was found; however, more than 70% of surveyed people referred to bad family support and more than 80% referred to stigma and discrimination in healthcare services because of their disease. It is well known that people with good family support have more probabilities of having regularity in medical controls and adherence to antiretroviral treatment [33, 34]. In contrast, qualitative reports referred that many young Awajun people living with HIV were thrown out of their houses or have relatives who speak ill of them, which favors the isolation of affected people [30].

Absence of family members or a partner could be a factor that decreases resilience to adverse situations, such as a HIV infection [35]. On the other hand, stigma and discrimination can be structural and manifested in institutionalized or social practices, both undermine availability, access, or use of health services, and prevent people from having a safe and protective family and community environment [36]. There is evidence that people living with HIV and indigenous people, independently, are victims of stigma and discrimination in healthcare services [31, 37], but only journalistic reports suggested that these factors could be determinants of leaving therapy or even hiding their diagnosis until days before death [30]. Future studies, especially with mixed methods approach, could be useful in demonstrating possible relationships, independently of this study’s findings.

There were no association between clinical features and ART adherence, but is important to note that almost 20% of surveyed individuals were identified as members of risk groups: men who have sex with men (MSM) (16.8%) and transgender people (1.9%). These findings are consistent with previous studies that have reported a high risk of sexually transmitted diseases or HIV in indigenous MSM [38]. Additionally, Bartlett et al. (2008) reported in previous research on four communities from the same region as the present study that all indigenous men who tested positive for HIV were from the MSM risk group, which represents 20% of total individuals living with HIV [39].

One limitation of this study is the inability to carry out a differentiated analysis by ethnicity, due to the small sample size of Wampis people and “others,“ who typically define themselves as “descendants of the union between a Wampis person and an Awajun person.“ Future research should better define this population group that continues to identify as part of an “indigenous or native population” despite being the product of the union between members of different communities and analyze their health problems.

Other limitations include the fact that the questionnaire did not include other components related to the opinions or attitudes of indigenous people about ART treatment, regardless of their adherence to it. Laura et al. (2021) found that there were some cases where ART treatment boxes were returned to health professionals so that they could give them to others who needed them more [30].

It is important to note that the methodological design of the research does not allow for the establishment of causal relationships, so future research should evaluate the true magnitude of the associations found. Additionally, the sample was obtained through a non-probabilistic method, so the results cannot be generalized to all of Peru.

The most significant strength of this research is its collaborative nature between researchers, regional health managers, and participating communities, from planning to execution. This allows for the short-term implementation of improvements in the ART program in these populations and emphasizes the importance of maintaining a constant intercultural dialogue between indigenous communities and decision-makers to quickly detect difficulties that may affect adherence to treatment.

## Conclusion

Only a third of participants reported complete adherence to antiretroviral therapy. Factors associated with adherence to antiretroviral medication were related to occupational status and reporting adverse reactions to the therapeutic scheme. Interventions aimed at improving adherence in indigenous people living with HIV should take these factors into consideration in order to develop effective implementation strategies.

## Supplementary Information

**Additional file 1.**

## Data Availability

The data that support the findings of this study are available from Instituto Nacional de Salud (Peru) but restrictions apply to the availability of these data, which were used under license for the current study, and so are not publicly available. Data are however available from the authors upon reasonable request to Félix Valenzuela Oré (fvalenzuelao_12@yahoo.es) and with permission of Instituto Nacional de Salud (Peru).

## Abbreviations

ART: Antiretroviral treatment
HIV: Human immunodeficiency virus
INEI: Instituto Nacional de Estadística e Informática – Perú
SMAQ: Simplified Medication Adherence Questionnaire
WHO: World Health Organization
