# Supplementary material for: Factors influencing adherence to anti-retroviral therapy in amazonian indigenous people living with HIV/AIDS
Source: BMC Public Health. 2023 Mar 15;23:497. doi: 10.1186/s12889-023-15362-y (PMC10015934; doi:10.1186/s12889-023-15362-y)
Supplement: Supplementary file 1 — Additional file 1. [file 12889_2023_15362_MOESM1_ESM.docx]

**Supplementary Material**

1. **Data collection form (English translation)**

| **ADHERENCE TO ART TREATMENT IN PEOPLE LIVING WITH HIV/AIDS IN**  **THE NATIVE COMMUNITIES AWAJUN AND WAMPIS - AMAZON**  **SURVEY INSTRUMENT** | | | | | | | | | | | | | | | | | | | | | | | | | | | | | | | | | | | |
| --- | --- | --- | --- | --- | --- | --- | --- | --- | --- | --- | --- | --- | --- | --- | --- | --- | --- | --- | --- | --- | --- | --- | --- | --- | --- | --- | --- | --- | --- | --- | --- | --- | --- | --- | --- |
|  |  |  |  |  |  |  |  |  |  |  |  | |  | |  | |  | |  | |  | |  | |  | |  | |  | |  | |  | |  |
|  |  |  |  |  |  |  |  |  |  |  |  | | Participant code: | | | | | | | | | |  | |  | |  | |  | |  | |  | |  |
|  |  |  |  |  |  |  |  |  |  |  |  | | Interviewer code: | | | | | | | | | | | |  | |  | |  | |  | |  | |  |
| Native community to which he/she belongs: | | | | | | | | | |  |  | |  | |  | |  | |  | |  | |  | |  | |  | |  | |  | |  | |  |
| Health center: | | | | | | |  |  |  |  |  | |  | |  | |  | |  | |  | |  | |  | |  | |  | |  | |  | |  |
| Health network: | | | | | | | |  |  |  |  | |  | |  | |  | |  | |  | |  | |  | |  | |  | |  | |  | |  |
| Date of data collection: ______ /______ /_______ | | | | | | | | | | | | | | | | | |  | |  | |  | |  | |  | |  | |  | |  | |  | |
|  |  |  |  |  |  |  |  |  |  |  |  | |  | |  | |  | |  | |  | |  | |  | |  | |  | |  | |  | |  |
| Good morning/afternoon, we are asking some questions to find out your opinion in relation to ART, your answers will help us to contribute to the improvement of health care, so please be as honest as possible in answering. | | | | | | | | | | | | | | | | | | | | | | | | | | | | | | | | | | | |
|  |  |  |  |  |  |  |  |  |  |  |  | |  | |  | |  | |  | |  | |  | |  | |  | |  | |  | |  | |  |
|  | **I: Personal characteristics** | | | | | | | | |  |  | |  | |  | |  | |  | |  | |  | |  | |  | |  | |  | |  | |  |
|  |  | 1. | Etnia | |  |  |  |  |  |  |  | |  | |  | |  | |  | |  | |  | |  | |  | |  | |  | |  | |  |
|  |  |  |  | 1. | Awajún | |  |  |  |  |  | |  | |  | |  | |  | |  | |  | |  | |  | |  | |  | |  | |  |
|  |  |  |  | 2. | Wampis | | |  |  |  |  | |  | |  | |  | |  | |  | |  | |  | |  | |  | |  | |  | |  |
|  |  |  |  | 3. | Others (specify) ………....…...…………..…………….. | | | | | | | | | | | | | | |  | |  | |  | |  | |  | |  | |  | |  | |
|  |  |  |  |  |  |  |  |  |  |  |  | |  | |  | |  | |  | |  | |  | |  | |  | |  | |  | |  | |  |
|  |  | 2. | Age (in years-old): | | | |  |  |  |  |  | |  | |  | |  | |  | |  | |  | |  | |  | |  | |  | |  | |  |
|  |  | 3. | Gender | |  |  |  |  |  |  |  | |  | |  | |  | |  | |  | |  | |  | |  | |  | |  | |  | |  |
|  |  |  |  | 1. | Female | | |  |  |  |  | |  | |  | |  | |  | |  | |  | |  | |  | |  | |  | |  | |  |
|  |  |  |  | 2. | Male | | |  |  |  |  | |  | |  | |  | |  | |  | |  | |  | |  | |  | |  | |  | |  |
|  |  |  |  |  |  |  |  |  |  |  |  | |  | |  | |  | |  | |  | |  | |  | |  | |  | |  | |  | |  |
|  |  | 4. | Civil status | | |  |  |  |  |  |  | |  | |  | |  | |  | |  | |  | |  | |  | |  | |  | |  | |  |
|  |  |  |  | 1. | Married | | |  |  |  |  | |  | |  | |  | |  | |  | |  | |  | |  | |  | |  | |  | |  |
|  |  |  |  | 2. | Single | | |  |  |  |  | |  | |  | |  | |  | |  | |  | |  | |  | |  | |  | |  | |  |
|  |  |  |  | 3. | With partner, not married | | | | | |  | |  | |  | |  | |  | |  | |  | |  | |  | |  | |  | |  | |  |
|  |  |  |  | 4. | Divorced | | | |  |  |  | |  | |  | |  | |  | |  | |  | |  | |  | |  | |  | |  | |  |
|  |  |  |  | 5. | Widower | | |  |  |  |  | |  | |  | |  | |  | |  | |  | |  | |  | |  | |  | |  | |  |
|  |  |  |  |  |  |  |  |  |  |  |  | |  | |  | |  | |  | |  | |  | |  | |  | |  | |  | |  | |  |
|  |  | 5. | Educational level: | | | | | |  |  |  | |  | |  | |  | |  | |  | |  | |  | |  | |  | |  | |  | |  |
|  |  |  |  | 1. | No studies | | | | | | |  | |  | |  | |  | |  | |  | |  | |  | |  | |  | |  | |  | |
|  |  |  |  | 2. | Primary level | | | | |  |  | |  | |  | |  | |  | |  | |  | |  | |  | |  | |  | |  | |  |
|  |  |  |  | 3. | Secundary level | | | | |  |  | |  | |  | |  | |  | |  | |  | |  | |  | |  | |  | |  | |  |
|  |  |  |  | 4. | Universitary or technical studies | | | | | | | | | | | | | |  | |  | |  | |  | |  | |  | |  | |  | |  |
|  |  |  |  |  |  |  |  |  |  |  |  | |  | |  | |  | |  | |  | |  | |  | |  | |  | |  | |  | |  |
|  |  | 6. | Occupation (at the moment to be surveyed) | | | | | | | | | | | | | | | | | | | | | | | | |  | |  | |  | |  | |
|  |  |  |  | 1. | Agriculture | | |  |  |  |  | |  | |  | |  | |  | |  | |  | |  | |  | |  | |  | |  | |  |
|  |  |  |  | 2. | Hunting-fishing | | | | | |  | |  | |  | |  | |  | |  | |  | |  | |  | |  | |  | |  | |  |
|  |  |  |  | 3. | Worker | |  |  |  |  |  | |  | |  | |  | |  | |  | |  | |  | |  | |  | |  | |  | |  |
|  |  |  |  | 4. | Employee | | |  |  |  |  | |  | |  | |  | |  | |  | |  | |  | |  | |  | |  | |  | |  |
|  |  |  |  | 5. | Student | | |  |  |  |  | |  | |  | |  | |  | |  | |  | |  | |  | |  | |  | |  | |  |
|  |  |  |  | 6. | Other occupations | | | | | | | |  | |  | |  | |  | |  | |  | |  | |  | |  | |  | |  | |  |
|  |  |  |  |  |  |  |  |  |  |  |  | |  | |  | |  | |  | |  | |  | |  | |  | |  | |  | |  | |  |

|  |  |  |  |  |  |  |  |  |  |  |  |  |  |  |  |  |  |  |  |  |  |  |
| --- | --- | --- | --- | --- | --- | --- | --- | --- | --- | --- | --- | --- | --- | --- | --- | --- | --- | --- | --- | --- | --- | --- |
|  |  | 7. | What are your religion? | | | | | | | |  |  |  |  |  |  |  |  |  |  |  |  |
|  |  |  |  | 1. | Catholic | | |  |  |  |  |  |  |  |  |  |  |  |  |  |  |  |
|  |  |  |  | 2. | Evangelical (specify: ……………………………………) | | | | | | | | | | |  |  |  |  |  |  |  |
|  |  |  |  | 3. | Christian | | |  |  |  |  |  |  |  |  |  |  |  |  |  |  |  |
|  |  |  |  | 4. | Israelite | | | |  |  |  |  |  |  |  |  |  |  |  |  |  |  |
|  |  |  |  | 5. | Other | |  |  |  |  |  |  |  |  |  |  |  |  |  |  |  |  |
|  |  |  |  | 6. | No religión | | |  |  |  |  |  |  |  |  |  |  |  |  |  |  |  |
|  |  |  |  |  |  |  |  |  |  |  |  |  |  |  |  |  |  |  |  |  |  |  |
|  |  | 8. | How much is your mensual income? (PEN) | | | | | | | | | | | | |  |  |  | | |  |  |
|  |  |  |  |  |  |  |  |  |  |  |  |  |  |  |  |  |  |  |  |  |  |  |
|  |  | 9. | Do you drink alcoholic beverages at least once a week? | | | | | | | | | | | | | | |  |  |  |  |  |
|  |  |  |  | 1. | Yes |  |  |  |  |  |  |  |  |  |  |  |  |  |  |  |  |  |
|  |  |  |  | 2. | Not |  |  |  |  |  |  |  |  |  |  |  |  |  |  |  |  |  |
|  |  |  |  |  |  |  |  |  |  |  |  |  |  |  |  |  |  |  |  |  |  |  |
|  |  | 10. | Do you use tobacco (cigars or cigarettes) at least once a week? | | | | | | | | | | | | | | | | |  |  |  |
|  |  |  |  | 1. | Yes |  |  |  |  |  |  |  |  |  |  |  |  |  |  |  |  |  |
|  |  |  |  | 2. | Not |  |  |  |  |  |  |  |  |  |  |  |  |  |  |  |  |  |
|  |  |  |  |  |  |  |  |  |  |  |  |  |  |  |  |  |  |  |  |  |  |  |
|  |  | 11. | Do you use drugs or psychoactive substances at least once a week? | | | | | | | | | | | | | | | | |  |  |  |
|  |  |  |  | 1. | Yes |  |  |  |  |  |  |  |  |  |  |  |  |  |  |  |  |  |
|  |  |  |  | 2. | Not |  |  |  |  |  |  |  |  |  |  |  |  |  |  |  |  |  |
|  |  |  |  |  |  |  |  |  |  |  |  |  |  |  |  |  |  |  |  |  |  |  |
|  |  | 12. | Who do you live with? | | | | |  |  |  |  |  |  |  |  |  |  |  |  |  |  |  |
|  |  |  |  | 1. | With family | | |  |  |  |  |  |  |  |  |  |  |  |  |  |  |  |
|  |  |  |  | 2. | Alone and far to her/his family | | | | | | | |  |  |  |  |  |  |  |  |  |  |
|  |  |  |  | 3. | Alone and close to her/his familiy | | | | | | | |  |  |  |  |  |  |  |  |  |  |
|  |  |  |  |  |  |  |  |  |  |  |  |  |  |  |  |  |  |  |  |  |  |  |
|  |  | 13. | Do you often travel to cities? | | | | | | |  |  |  |  |  |  |  |  |  |  |  |  |  |
|  |  |  |  | 1. | Yes |  |  |  |  |  |  |  |  |  |  |  |  |  |  |  |  |  |
|  |  |  |  | 2. | Not |  |  |  |  |  |  |  |  |  |  |  |  |  |  |  |  |  |
|  |  |  |  |  |  |  |  |  |  |  |  |  |  |  |  |  |  |  |  |  |  |  |
|  | **II: Clinical characteristics** | | | | | | | | | | | | | | | |  |  |  |  |  |  |
|  |  | 14. | Risk population* | | | | |  |  |  |  |  |  |  |  |  |  |  |  |  |  |  |
|  |  |  |  | 1. | MSM | |  |  |  |  |  |  |  |  |  |  |  |  |  |  |  |  |
|  |  |  |  | 2. | TG |  |  |  |  |  |  |  |  |  |  |  |  |  |  |  |  |  |
|  |  |  |  | 3. | SW |  |  |  |  |  |  |  |  |  |  |  |  |  |  |  |  |  |
|  |  |  |  | 4. | GP |  |  |  |  |  |  |  |  |  |  |  |  |  |  |  |  |  |
|  |  |  |  |  |  |  |  |  |  |  |  |  |  |  |  |  |  |  |  |  |  |  |
|  |  | 15. | HIV/AIDS diagnosis date | | | | | | | |  |  |  |  |  |  |  |  |  |  |  |  |
|  |  |  |  |  |  |  |  |  |  |  |  |  |  |  |  |  |  |  |  |  |  |  |
|  |  | 16. | Are opportunistic diseases reported?** | | | | | | | | |  |  |  |  |  |  |  |  |  |  |  |
|  |  |  |  | 1. | Yes |  |  |  |  |  |  |  |  |  |  |  |  |  |  |  |  |  |
|  |  |  |  | 2. | Not |  |  |  |  |  |  |  |  |  |  |  |  |  |  |  |  |  |
|  |  |  |  |  |  |  |  |  |  |  |  |  |  |  |  |  |  |  |  |  |  |  |
|  |  | 17. | Clinical stage | | | |  |  |  |  |  |  |  |  |  |  |  |  |  |  |  |  |
|  |  |  |  | 1. | EC1 | |  |  |  |  |  |  |  |  |  |  |  |  |  |  |  |  |
|  |  |  |  | 2. | EC2 | |  |  |  |  |  |  |  |  |  |  |  |  |  |  |  |  |
|  |  |  |  | 3. | EC3 | |  |  |  |  |  |  |  |  |  |  |  |  |  |  |  |  |
|  |  |  |  | 4. | EC4 | |  |  |  |  |  |  |  |  |  |  |  |  |  |  |  |  |
|  |  |  |  |  |  |  |  |  |  |  |  |  |  |  |  |  |  |  |  |  |  |  |

|  |  | |  | |  | |  | |  |  | |  | |  | |  | |  | |  | |  | |  | |  | |  | |  | |  | |  | |  | |  | |  | |  | |  |
| --- | --- | --- | --- | --- | --- | --- | --- | --- | --- | --- | --- | --- | --- | --- | --- | --- | --- | --- | --- | --- | --- | --- | --- | --- | --- | --- | --- | --- | --- | --- | --- | --- | --- | --- | --- | --- | --- | --- | --- | --- | --- | --- | --- | --- |
|  |  | | 18. | | TB co-infection | | | | | | | | |  | |  | |  | |  | |  | |  | |  | |  | |  | |  | |  | |  | |  | |  | |  | |  |
|  |  | |  | |  | | 1. | | Yes |  | |  | |  | |  | |  | |  | |  | |  | |  | |  | |  | |  | |  | |  | |  | |  | |  | |  |
|  |  | |  | |  | | 2. | | Not |  | |  | |  | |  | |  | |  | |  | |  | |  | |  | |  | |  | |  | |  | |  | |  | |  | |  |
|  |  | | 19. | | HvB co-infection | | | | | | | | | | |  | |  | |  | |  | |  | |  | |  | |  | |  | |  | |  | |  | |  | |  | |  |
|  |  | |  | |  | | 1. | | Yes |  | |  | |  | |  | |  | |  | |  | |  | |  | |  | |  | |  | |  | |  | |  | |  | |  | |  |
|  |  | |  | |  | | 2. | | Not |  | |  | |  | |  | |  | |  | |  | |  | |  | |  | |  | |  | |  | |  | |  | |  | |  | |  |
|  |  | |  | |  | |  | |  |  | |  | |  | |  | |  | |  | |  | |  | |  | |  | |  | |  | |  | |  | |  | |  | |  | |  |
|  | **III: ART related characteristics** | | | | | | | | | | | | | | | | | | | | | | | | | | | | | | | | |  | |  | |  | |  | |  | |  |
|  |  | | 20. | | Therapeutic schema | | | | | | | | | | | | | | |  | |  | |  | |  | |  | |  | |  | |  | |  | |  | |  | |  | |  |
|  |  | |  | |  | | 1. | | First line | | | | | | | | | | | | |  | |  | |  | |  | |  | |  | |  | |  | |  | |  | |  | |  |
|  |  | |  | |  | | 2. | | Tenofovir-based | | | | | | | | | | | | |  | |  | |  | |  | |  | |  | |  | |  | |  | |  | |  | |  |
|  |  | |  | |  | | 3. | | Abacavir-based | | | | | | | | | | | | |  | |  | |  | |  | |  | |  | |  | |  | |  | |  | |  | |  |
|  |  | |  | |  | | 4. | | Zidovudine-based | | | | | | | | | | | | | | |  | |  | |  | |  | |  | |  | |  | |  | |  | |  | |  |
|  |  | |  | |  | | 5. | | Unknown | | | | | | | | | | |  | |  | |  | |  | |  | |  | |  | |  | |  | |  | |  | |  | |  |
|  |  | |  | |  | |  | |  |  | |  | |  | |  | |  | |  | |  | |  | |  | |  | |  | |  | |  | |  | |  | |  | |  | |  |
|  |  | | 21. | | ART starting date | | | | | | | | | | | | | | | | | | | | | | |  | |  | |  | |  | |  | |  | |  | |  | |  |
|  |  | |  | |  | |  | |  |  | |  | |  | |  | |  | |  | |  | |  | |  | |  | |  | |  | |  | |  | |  | |  | |  | |  |
|  |  | | 22. | | The treatment does you follow for HIV is: | | | | | | | | | | | | | | | | | | | | |  | |  | |  | |  | |  | |  | |  | |  | |  | |  |
|  |  | |  | |  | | 1. | | Only with prescribed medications | | | | | | | | | | | | | | |  | |  | |  | |  | |  | |  | |  | |  | |  | |  | |  |
|  |  | |  | |  | | 2. | | Only with medicinal plants | | | | | | | | | | | | |  | |  | |  | |  | |  | |  | |  | |  | |  | |  | |  | |  |
|  |  | |  | |  | | 3. | | With medicines and medicinal plants at the same time | | | | | | | | | | | | | | | | | | | | | | |  | |  | |  | |  | |  | |  | |  |
|  |  | |  | |  | | 4. | | Self medication | | | | | | |  | |  | |  | |  | |  | |  | |  | |  | |  | |  | |  | |  | |  | |  | |  |
|  |  | |  | |  | |  | |  |  | |  | |  | |  | |  | |  | |  | |  | |  | |  | |  | |  | |  | |  | |  | |  | |  | |  |
|  |  | | 23. | | Presence of adverse reactions to ART | | | | | | | | | | | | | | | | | | |  | |  | |  | |  | |  | |  | |  | |  | |  | |  | |  |
|  |  | |  | |  | | 1. | | Yes |  | |  | |  | |  | |  | |  | |  | |  | |  | |  | |  | |  | |  | |  | |  | |  | |  | |  |
|  |  | |  | |  | | 2. | | Not |  | |  | |  | |  | |  | |  | |  | |  | |  | |  | |  | |  | |  | |  | |  | |  | |  | |  |
|  |  | |  | |  | |  | |  |  | |  | |  | |  | |  | |  | |  | |  | |  | |  | |  | |  | |  | |  | |  | |  | |  | |  |
|  | **IV: Socio-cultural characteristics** | | | | | | | | | | | | | | | | | | |  | |  | |  | |  | |  | |  | |  | |  | |  | |  | |  | |  | |  |
|  |  | | 24. | | What is the alternative that best matches your opinion about the cause of HIV/AIDS | | | | | | | | | | | | | | | | | | | | | | | | | | | | | | | | | | | | | | |  |
|  |  | |  | |  | | 1. | | A sexually transmitted or bloodborne disease | | | | | | | | | | | | | | | | | | | | | | |  | |  | |  | |  | |  | |  | |  |
|  |  | |  | |  | | 2. | | A disease produced by witchcraft | | | | | | | | | | | | | | | | |  | |  | |  | |  | |  | |  | |  | |  | |  | |  |
|  |  | |  | |  | | 3. | | God's punishment | | | | | | | | | | | | |  | |  | |  | |  | |  | |  | |  | |  | |  | |  | |  | |  |
|  |  | |  | |  | | 4. | | Strange disease transmitted by external others | | | | | | | | | | | | | | | | | | | | | | |  | |  | |  | |  | |  | |  | |  |
|  |  | |  | |  | | 5. | | Other causes | | | | | | |  | |  | |  | |  | |  | |  | |  | |  | |  | |  | |  | |  | |  | |  | |  |
|  |  | |  | |  | |  | |  |  | |  | |  | |  | |  | |  | |  | |  | |  | |  | |  | |  | |  | |  | |  | |  | |  | |  |
|  |  | | 25. | | How would you rate the support and support you receive from your family regarding the continuity of your treatment with ART? | | | | | | | | | | | | | | | | | | | | | | | | | | | | | | | | | | | | |  | |  |
|  |  | |  | |  |  |  |  |  |  |  |  |  |  |  |  |  |  |  |  |  |  |  |  |  |  |  |  |  |  |  |  |  |  |  |  |  |  |  |  |  |  | |  |
|  |  | |  | |  | | 1. | | Very Good | | | | |  | |  | |  | |  | |  | |  | |  | |  | |  | |  | |  | |  | |  | |  | |  | |  |
|  |  | |  | |  | | 2. | | Good | | |  | |  | |  | |  | |  | |  | |  | |  | |  | |  | |  | |  | |  | |  | |  | |  | |  |
|  |  | |  | |  | | 3. | | Regular | | |  | |  | |  | |  | |  | |  | |  | |  | |  | |  | |  | |  | |  | |  | |  | |  | |  |
|  |  | |  | |  | | 4. | | Bad | | |  | |  | |  | |  | |  | |  | |  | |  | |  | |  | |  | |  | |  | |  | |  | |  | |  |
|  |  | |  | |  | | 5. | | Very bad | | | | |  | |  | |  | |  | |  | |  | |  | |  | |  | |  | |  | |  | |  | |  | |  | |  |
|  |  | |  | |  | |  | |  |  | |  | |  | |  | |  | |  | |  | |  | |  | |  | |  | |  | |  | |  | |  | |  | |  | |  |
|  |  | | 26. | | In general, do you believe that you will be treated the same as other people when you go to the health facility, due to your condition? | | | | | | | | | | | | | | | | | | | | | | | | | | | | | | | | | | | | |  | |  |
|  |  | |  | |  |  |  |  |  |  |  |  |  |  |  |  |  |  |  |  |  |  |  |  |  |  |  |  |  |  |  |  |  |  |  |  |  |  |  |  |  |  | |  |
|  |  | |  | |  | | 1. | | Yes |  | |  | |  | |  | |  | |  | |  | |  | |  | |  | |  | |  | |  | |  | |  | |  | |  | |  |
|  |  | |  | |  | | 2. | | Not |  | |  | |  | |  | |  | |  | |  | |  | |  | |  | |  | |  | |  | |  | |  | |  | |  | |  |
|  |  | |  | |  | |  | |  |  | |  | |  | |  | |  | |  | |  | |  | |  | |  | |  | |  | |  | |  | |  | |  | |  | |  |
|  |  | | 27. | | Since you received treatment, do you have considered that care at the health facility is normal, due to your condition? | | | | | | | | | | | | | | | | | | | | | | | | | | | | | | | | | | | | |  | |  |
|  |  | |  | |  |  |  |  |  |  |  |  |  |  |  |  |  |  |  |  |  |  |  |  |  |  |  |  |  |  |  |  |  |  |  |  |  |  |  |  |  |  | |  |
|  |  | |  | |  | | 1. | | Yes |  | |  | |  | |  | |  | |  | |  | |  | |  | |  | |  | |  | |  | |  | |  | |  | |  | |  |
|  |  | |  | |  | | 2. | | Not |  | |  | |  | |  | |  | |  | |  | |  | |  | |  | |  | |  | |  | |  | |  | |  | |  | |  |
|  | **V: Characteristics related to access to health services** | | | | | | | | | | | | | | | | | | | | | | | | | | | | | | | | | |  | |  | |  | |  | |  | |
|  |  | 28. | | What is the health insurance do you have? | | | | | | | | | | | | | | | | | | | | | | | | | | | | |  | |  | |  | |  | |  | |  | |
|  |  |  | |  | | 1. | | None | | | | | | |  | |  | |  | |  | |  | |  | |  | |  | |  | |  | |  | |  | |  | |  | |  | |
|  |  |  | |  | | 2. | | SIS | | |  | |  | |  | |  | |  | |  | |  | |  | |  | |  | |  | |  | |  | |  | |  | |  | |  | |
|  |  |  | |  | | 3. | | EsSalud | | | | |  | |  | |  | |  | |  | |  | |  | |  | |  | |  | |  | |  | |  | |  | |  | |  | |
|  |  |  | |  | | 4. | | Private insurance | | | | | | | | |  | |  | |  | |  | |  | |  | |  | |  | |  | |  | |  | |  | |  | |  | |
|  |  |  | |  | | 5. | | National police | | | | | | | | | | | | |  | |  | |  | |  | |  | |  | |  | |  | |  | |  | |  | |  | |
|  |  |  | |  | | 6. | | Armed forces | | | | | | | | | | |  | |  | |  | |  | |  | |  | |  | |  | |  | |  | |  | |  | |  | |
|  |  |  | |  | |  | |  | | |  | |  | |  | |  | |  | |  | |  | |  | |  | |  | |  | |  | |  | |  | |  | |  | |  | |
|  |  | 29. | | How long does it take to go to the nearest health center? (mins) | | | | | | | | | | | | | | | | | | | | | | | | | | | | |  | |  | |  | |  | |  | |  | |
|  |  |  | |  | |  | |  | | |  | |  | |  | |  | |  | |  | |  | |  | |  | |  | |  | |  | |  | |  | |  | |  | |  | |
|  |  | 30. | | How much money (in soles) do you spend per month to travel to the health center? | | | | | | | | | | | | | | | | | | | | | | | | | | | | | | | | | | | | | | | | |
|  |  |  | |  | |  | |  | | |  | |  | |  | |  | |  | |  | |  | |  | |  | |  | |  | |  | |  | | | | | |  | |  | |
|  |  |  | |  | |  | |  | | |  | |  | |  | |  | |  | |  | |  | |  | |  | |  | |  | |  | |  | |  | |  | |  | |  | |
|  |  | 31. | | How much money (in soles) do you spend per month on treatment for your condition? | | | | | | | | | | | | | | | | | | | | | | | | | | | | | | | | | | | | |  | |  | |
|  |  |  | |  | |  | |  | | |  | |  | |  | |  | |  | |  | |  | |  | |  | |  | |  | |  | |  | | | | | |  | |  | |
|  |  |  | |  | |  | |  | | |  | |  | |  | |  | |  | |  | |  | |  | |  | |  | |  | |  | |  | |  | |  | |  | |  | |
|  | **VI: TAR adherence (*Simplified medication adherence questionnaire*-SMAQ)** | | | | | | | | | | | | | | | | | | | | | | | | | | | | | | | | | | | | | |  | |  | |  | |
|  |  | 32. | | Do you ever forget to take your medication? | | | | | | | | | | | | | | | | | | | | |  | |  | |  | |  | |  | |  | |  | |  | |  | |  | |
|  |  |  | |  | | 1. | | Yes | | |  | |  | |  | |  | |  | |  | |  | |  | |  | |  | |  | |  | |  | |  | |  | |  | |  | |
|  |  |  | |  | | 2. | | Not | | |  | |  | |  | |  | |  | |  | |  | |  | |  | |  | |  | |  | |  | |  | |  | |  | |  | |
|  |  |  | |  | |  | |  | | |  | |  | |  | |  | |  | |  | |  | |  | |  | |  | |  | |  | |  | |  | |  | |  | |  | |
|  |  | 33. | | Did you always take your medications at the right time? | | | | | | | | | | | | | | | | | | | | | | | | | | | | | | |  | |  | |  | |  | |  | |
|  |  |  | |  | | 1. | | Yes | | |  | |  | |  | |  | |  | |  | |  | |  | |  | |  | |  | |  | |  | |  | |  | |  | |  | |
|  |  |  | |  | | 2. | | Not | | |  | |  | |  | |  | |  | |  | |  | |  | |  | |  | |  | |  | |  | |  | |  | |  | |  | |
|  |  |  | |  | |  | |  | | |  | |  | |  | |  | |  | |  | |  | |  | |  | |  | |  | |  | |  | |  | |  | |  | |  | |
|  |  | 34. | | Do you ever stop taking your medicine if you feel sick? | | | | | | | | | | | | | | | | | | | | | | | | | | | | | | |  | |  | |  | |  | |  | |
|  |  |  | |  | | 1. | | Yes | | |  | |  | |  | |  | |  | |  | |  | |  | |  | |  | |  | |  | |  | |  | |  | |  | |  | |
|  |  |  | |  | | 2. | | Not | | |  | |  | |  | |  | |  | |  | |  | |  | |  | |  | |  | |  | |  | |  | |  | |  | |  | |
|  |  |  | |  | |  | |  | | |  | |  | |  | |  | |  | |  | |  | |  | |  | |  | |  | |  | |  | |  | |  | |  | |  | |
|  |  | 35. | | Did you forget to take your medication over the weekend? | | | | | | | | | | | | | | | | | | | | | | | | | | |  | |  | |  | |  | |  | |  | |  | |
|  |  |  | |  | | 1. | | Yes | | |  | |  | |  | |  | |  | |  | |  | |  | |  | |  | |  | |  | |  | |  | |  | |  | |  | |
|  |  |  | |  | | 2. | | Not | | |  | |  | |  | |  | |  | |  | |  | |  | |  | |  | |  | |  | |  | |  | |  | |  | |  | |
|  |  |  | |  | |  | |  | | |  | |  | |  | |  | |  | |  | |  | |  | |  | |  | |  | |  | |  | |  | |  | |  | |  | |
|  |  | 36. | | In the last week, how many times did you not take medication doses? | | | | | | | | | | | | | | | | | | | | | | | | | | | | | | | | | | | | |  | |  | |
|  |  |  | |  | | 1. | | None | | | | | | |  | |  | |  | |  | |  | |  | |  | |  | |  | |  | |  | |  | |  | |  | |  | |
|  |  |  | |  | | 2. | | 1-2 | | |  | |  | |  | |  | |  | |  | |  | |  | |  | |  | |  | |  | |  | |  | |  | |  | |  | |
|  |  |  | |  | | 3. | | 3-5 | | |  | |  | |  | |  | |  | |  | |  | |  | |  | |  | |  | |  | |  | |  | |  | |  | |  | |
|  |  |  | |  | | 4. | | 6-10 | | | | |  | |  | |  | |  | |  | |  | |  | |  | |  | |  | |  | |  | |  | |  | |  | |  | |
|  |  |  | |  | | 5. | | More than 10 | | | | | | |  | |  | |  | |  | |  | |  | |  | |  | |  | |  | |  | |  | |  | |  | |  | |
|  |  |  | |  | |  | |  | | |  | |  | |  | |  | |  | |  | |  | |  | |  | |  | |  | |  | |  | |  | |  | |  | |  | |
|  |  | 37. | | In the last three months, how many days did you forget to take medication? | | | | | | | | | | | | | | | | | | | | | | | | | | | | | | | | | | |  | |  | |  | |
|  |  |  | |  | | 1. | | None | | | | | | |  | |  | |  | |  | |  | |  | |  | |  | |  | |  | |  | |  | |  | |  | |  | |
|  |  |  | |  | | 2. | | Less than two days | | | | | | | | | | |  | |  | |  | |  | |  | |  | |  | |  | |  | |  | |  | |  | |  | |
|  |  |  | |  | | 3. | | Two or more days | | | | | | | | |  | |  | |  | |  | |  | |  | |  | |  | |  | |  | |  | |  | |  | |  | |
|  |  |  | |  | | 4. | | Unknown | | | | | | | | | | | | |  | |  | |  | |  | |  | |  | |  | |  | |  | |  | |  | |  | |
|  |  |  | |  | |  | |  | | |  | |  | |  | |  | |  | |  | |  | |  | |  | |  | |  | |  | |  | |  | |  | |  | |  | |

* MSM: Men who have sex with men; TG: Transgender people; SW: Sexual workers; GP: General population; TB: Tuberculosis; HvB: Hepatitis B virus.

** Defined as any disease that occur more frequently and are more severe in people with HIV. Reference list in peruvian context: tuberculosis, hepatitis B, candidiasis, coccidioidomycosis, cryptococcosis, cryptosporidiosis, cytomegalovirus, HIV-related encephalopathy, herpes simplex virus, histoplasmosis, Kaposi’s sarcoma, *Mycobacterium avium* complex, pneumocystis pneumonia, toxoplasmosis,

1. **Therapeutic ART schemas by Ministry of Health (Peru)**

| **Type of therapeutic schema** | **Doses** | **Considerations** |
| --- | --- | --- |
| **First line** |  |  |
| Tenofovir 300 mg (TDF)/Emtricitabine 200 mg (FTC)/ Efavirenz 600 mg (EFV) | One tablet of TDF/FTC/EFV on fixed-dose combination (FDC) each 24 hours at bedtime | First election scheme |
| Tenofovir 300 mg (TDF)/Emtricitabine 200 mg (FTC) + Efavirenz 600 mg (EFV) | One tablet of TDF/FTC (FDC) + one tablet of EFV, each 24 hours at bedtime |  |
| Tenofovir 300 mg (TDF)/Lamivudine 150 mg (3TC)/ Efavirenz 600 mg (EFV) | One tablet of TDF + two tablets of 3TC + one tablet of EFV, all each 24 hours at bedtime | Used when previous presentations of fixed dose combination are not disponible. |
| **Tenofovir based (TDF)** |  |  |
| Tenofovir 300 mg (TDF)/Emtricitabine 200 mg (FTC) + Lopinavir/Ritonavir 200/50 mg (LPV/rtv) | One tablet of TDF/FTC (FDC) each 24 hours + two tablets of LPV/rtv each 12 hours. | There are used when Efavirenz were contraindicated or there was any history of adverse events related to efavirenz. |
| Tenofovir 300 mg (TDF) + Lamivudine 150 mg (3TC) + Lopinavir/Ritonavir 200/50 mg (LPV/rtv) | One tablet of TDF + two tablets of 3TC each 24 hours + two tablets of LPV/rtv each 12 hours. |  |
| Tenofovir 300 mg (TDF) + Lamivudine 150 mg (3TC) + Dolutegravir 50 mg (DTG) | One tablet of TDF + two tablets of 3TC + one tablet of DTG each 24 hours |  |
| Tenofovir 300 mg (TDF)/Emtricitabine 200 mg (FTC) + Dolutegravir 50 mg (DTG) | One tablet of TDF/FTC (FDC) each 24 hours + one tablet of DTG each 24 hours |  |
| **Abacavir based (ABC)** |  |  |
| Abacavir 600 mg (ABC)/Lamivudine 300 mg (3TC) + Efavirenz 600 mg (EFV) | One tablet of ABC/3TC (FDC) each 24 hours + one tablet of EFV at bedtime. | Only in patients with viral count < 100 000 copies/mL, with HLAB*5701 negative. |
| Abacavir 300 mg (ABC) + Lamivudine 150 mg (3TC) + Efavirenz 600 mg (EFV) | One tablet of ABC each 12 hours + one tablet of 3TC each 12 hours + one tablet of EFV each 24 hours at bedtime |  |
| Abacavir 600 mg (ABC)/Lamivudine 300 mg (3TC) + Dolutegavir 50 mg (DTG) | One tablet ABC/3TC each 24 hours + one tablet of DTG each 24 hours. | Used when Efavirenz were contraindicated or there was any history of adverse events related to efavirenz. |
| Abacavir 300 mg (ABC) + Lamivudine 150 mg (3TC) + Dolutegavir 50 mg (DTG) | One tablet of ABC + one tablet of 3TC each 12 hours + one tablet of DTG each 24 hours | Only in patients with HLAB*5701 negative, independently of viral count. |
| **Zidovudine based (AZT)** |  |  |
| Zidovudine 300 mg (AZT)/ Lamivudine 150 mg (3TC) + Efavirenz 600 mg (EFV) | One tablet of AZT/3TC (FDC) each 12 hours + one tablet of EFV each 24 hours at bedtime | Used when Tenofovir or Abacavir were contraindicated. |
| Zidovudine 300 mg (AZT)/ Lamivudine 150 mg (3TC)/Nevirapine 200 mg (NVP) | One tablet of AZT/3TC/NVP (FDC) each 12 hours | Used when Tenofovir or Abacavir and Efavirenz were contraindicated. Not for first time ART therapy |

Source: Norma Técnica de Salud de Atención Integral del Adulto con Infección por el Virus de la Inmunodeficiencia Humana. Norma Técnica de Salud N° 097-MINSA/2018/DGIESP-V.03 (R.M. 215-2018/MINSA). Ministry of Health (Peru).
